# ANCA-negative EGPA with migratory pulmonary masses: A case report of mepolizumab response and literature review

**DOI:** 10.1097/MD.0000000000047477

**Published:** 2026-01-30

**Authors:** Wenhua Gu, Wei Yang, Chaomin Wu, XiongBiao Wang

**Affiliations:** a Department of Pulmonary and Critical Care Medicine, Putuo Hospital, Shanghai University of Traditional Chinese Medicine, Shanghai, China.

**Keywords:** asthma, EGPA, eosinophilic granulomatosis with polyangiitis, mepolizumab, sinusitis

## Abstract

**Rationale:**

Eosinophilic granulomatosis with polyangiitis (EGPA) is a small- to medium-vessel vasculitis commonly associated with asthma and eosinophilia. Misdiagnosis as isolated asthma may delay recognition and treatment.

**Patient concerns:**

A patient with a long history of asthma presented with persistent respiratory symptoms and a pulmonary mass initially presumed to be asthma-related pathology.

**Diagnosis:**

Clinical evaluation, laboratory findings of eosinophilia, and imaging supported the diagnosis of EGPA rather than isolated asthma.

**Interventions:**

The patient received glucocorticoid therapy (methylprednisolone) combined with mepolizumab.

**Outcomes:**

Treatment resulted in rapid symptomatic improvement and radiological resolution of the pulmonary mass within 1 month.

**Lessons:**

This case highlights the diagnostic challenge of distinguishing EGPA from long-standing asthma and supports the role of targeted biologic therapy in achieving effective disease control.

## 1. Introduction

Eosinophilic granulomatosis with polyangiitis (EGPA) is a form of necrotizing vasculitis that primarily involves small- and medium-sized blood vessels. It is characterized by eosinophilia and granulomatous inflammation, and is typically defined by the triad of asthma, eosinophilia, and necrotizing vasculitis. Due to presenting different respiratory symptoms at early-stage, EGPA patient is often misdiagnosed as other respiratory disorders. Till recent application of anti-IL-5 antibodies, the treatment had not made progress for a long time.

## 2. Case report

**Figure 1.**
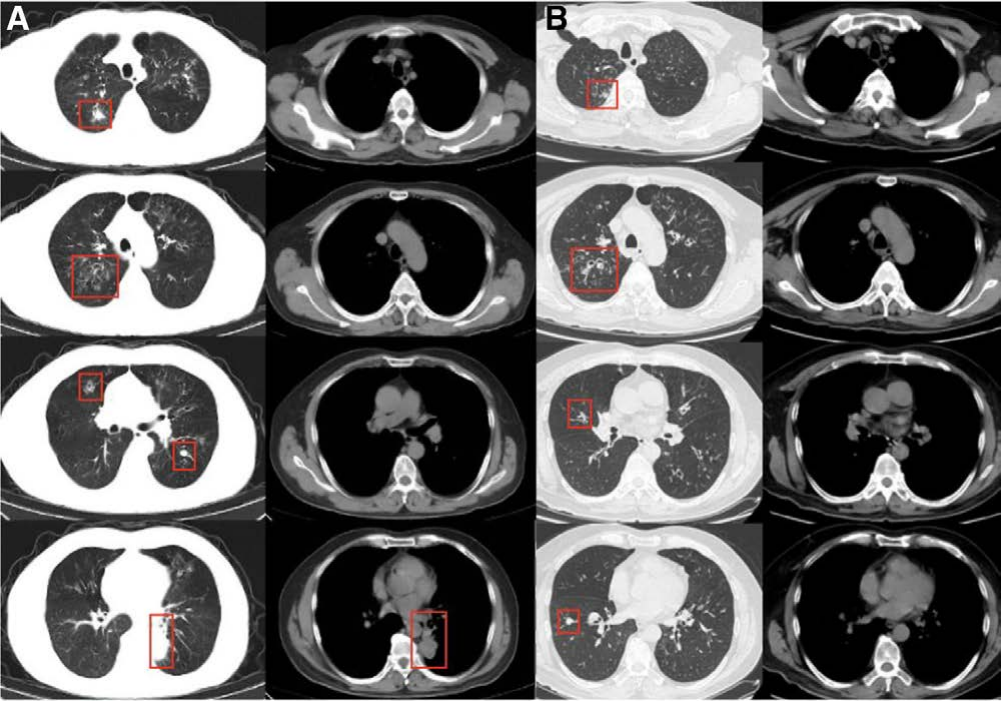
(A) CT findings of pulmonary lesions in November 2020: A mass was present in the left lower lobe. Bronchiectasis with infection affected the right upper and middle lobes and the left lung. (B) CT Findings of Pulmonary Lesions in March 2023: A nodule was located in the anterior basal segment of the right lower lobe. Multiple grain-sized nodules and opacities were seen in both upper lobes, with bronchiectasis in those lobes and the left lower lobe. CT = computed tomography.

A 56-year-old male patient complained of paroxysmal wheezing for 30 years, occurring at a frequency of 3 to 5 episodes per year. These episodes were not triggered by exercise or exposure to odors and were commonly accompanied by cough and expectoration. Elevated peripheral blood eosinophil counts (≥1 × 10^9^/L) were consistently observed across multiple laboratory assessments. The patient had been diagnosed with bronchial asthma at multiple medical institutions and had never experienced a severe acute exacerbation. The patient is a surgeon with no occupational exposure to agents linked to asthma or eosinophilia, such as organic dusts, industrial chemicals, fumes, or parasites. He also reports no significant environmental exposures, including mold, pets, or travel to endemic areas, and no use of medications or substances known to cause eosinophilic lung disease. During exacerbations, systemic glucocorticoids, aminophylline, and antibiotics were usually prescribed. Over the past decade, he had used Seretide (salmeterol xinafoate and fluticasone propionate inhalation powder) irregularly; however, he continued to experience recurrent acute episodes and required frequent use of inhaled salbutamol for symptom relief. In November 2020, the patient presented with worsening dyspnea, cough, and yellow sputum production. A chest computed tomography (CT) scan revealed a mass in the left lower lobe (Fig. 1). The patient was diagnosed with bronchial asthma complicated by pulmonary infection and received a 5-day course of prednisone and antibiotics, resulting in clinical improvement. One month later, the mass in the left lower lobe had resolved completely. A follow-up chest CT scan performed in March 2023 demonstrated multiple nodules located in the anterior basal segment of the right lower lobe. In September 2024, the patient developed exertional dyspnea in the absence of other symptoms such as cough, sputum production, chest pain, hemoptysis, fever, anorexia, or weight loss. Chest CT scan identified a newly emerged mass in the right upper lobe measuring approximately 55 × 41 mm, as well as diffuse bronchiectasis in both lungs (Fig. 4A). Given the suspicion of a malignant pulmonary neoplasm, the patient was admitted for further evaluation.

**Figure 2.**
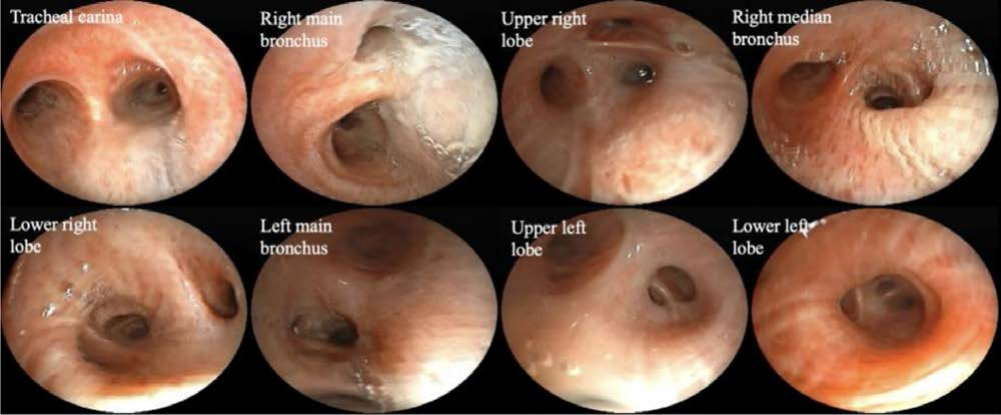
Electron bronchoscopy findings: The bronchial lumina on both sides are patent, with mucosal hypertrophy noted.

**Figure 3.**
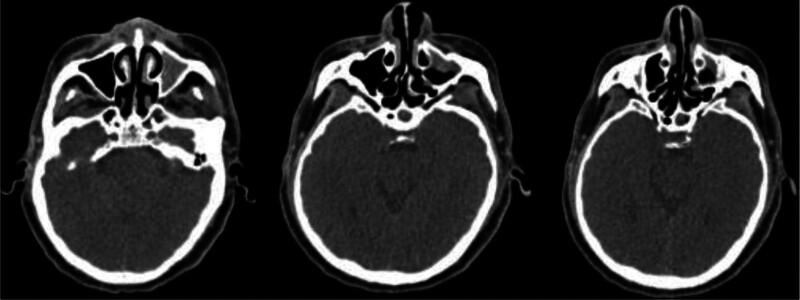
Paranasal sinus CT Findings in 2024: Multifocal inflammatory changes were observed across multiple sinus groups, along with a deviated nasal septum. CT = computed tomography.

Laboratory tests (September 2024): Complete blood count: White blood cell count: 11.7 × 10^9^/L, with differential: neutrophils 59.3%, lymphocytes 27.1%, monocytes 6.4%, eosinophils 6.5% (absolute count 760 × 10^6^/L), basophils 0.7%, Hemoglobin: 164 g/L, Platelet count: 278 × 10^9^/L, Eosinophil count: 760 × 10^6^/L. Liver and kidney functions, tumor markers were normal; Total IgE: >5000 kU/L; Autoantibodies: Cytoplasmic antineutrophil cytoplasmic antibody (c-ANCA): Negative (−), Anti-proteinase 3 antineutrophil cytoplasmic antibody (PR3-ANCA): 16 (<100 AU/mL), Perinuclear antineutrophil cytoplasmic antibody (p-ANCA): Negative (−), Myeloperoxidase antineutrophil cytoplasmic antibody (MPO-ANCA): 4 (<100 AU/mL); Serum cytokine levels: IL-2: 1.23 pg/mL, IL-4: 1.24 pg/mL, IL-5: 6.02 pg/mL, IL-6: 14.61 pg/mL. Bronchoscopy showed a patent airway with thickened mucosa but no neoplasia (Fig. 2). Histopathological analysis of the bronchial wall near the carina revealed mild papillary hyperplasia of the bronchial epithelium and eosinophilic infiltration. Bronchoalveolar lavage fluid (BALF) cell differential count showed predominant neutrophils (79%), lymphocytes (14%), eosinophils (5%), and monocytes (2%). Targeted next-generation sequencing (tNGS) identified Haemophilus influenzae and Pseudomonas aeruginosa. Pulmonary function tests revealed severe mixed ventilatory impairment with moderate reduction in diffusion capacity. Fractional exhaled nitric oxide (FeNO) was measured at 10 ppb. Sinus CT imaging demonstrated inflammatory involvement of multiple sinus groups (Fig. 3). Serological testing, including Aspergillus-specific IgE, anti-Aspergillus IgG, and galactomannan assay, yielded negative results. Electromyography showed no evidence of neurogenic or myogenic dysfunction. Resting transthoracic echocardiography demonstrated normal cardiac chamber sizes and preserved left ventricular systolic function (LVEF 67%), with mild diastolic dysfunction and no evidence of valvular disease, pulmonary hypertension, or pericardial effusion.

**Figure 4.**
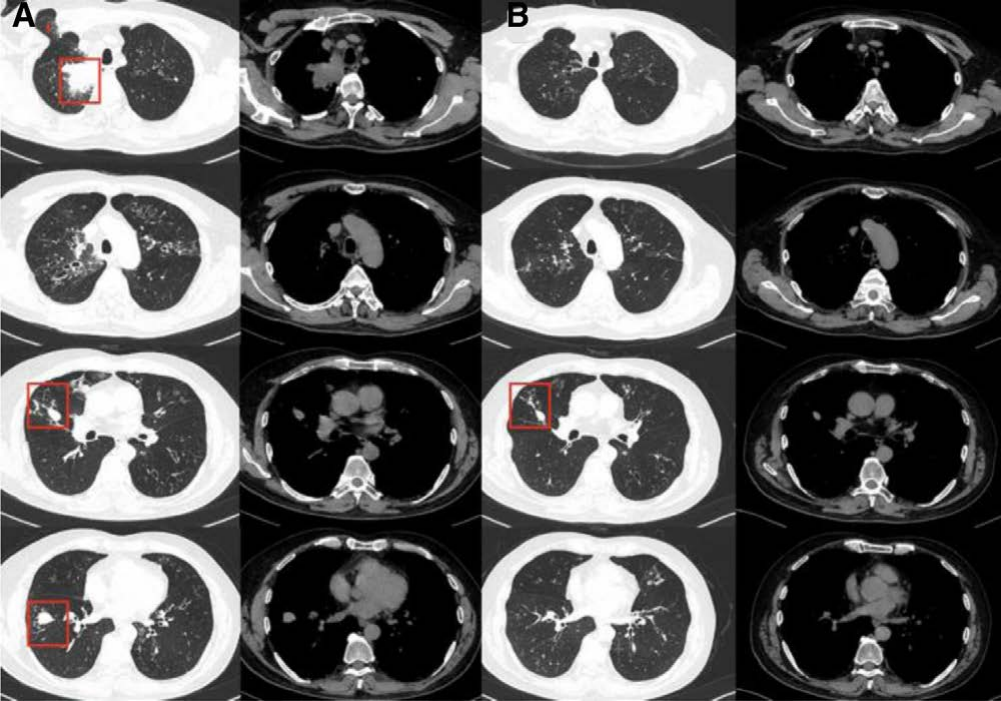
(A) CT Findings of Pulmonary Lesions in September 2024: A mass was seen in the right upper lobe. Bronchiectasis with a branching pattern involved the left lung, right upper lobe, and right middle lobe. Scattered patchy and micronodular opacities, as well as bilateral discrete nodules, were also present. (B) CT findings following treatment (October 2024): The right upper lobe lesion had resolved completely compared to the September 2024 findings. CT = computed tomography.

**Figure 5.**
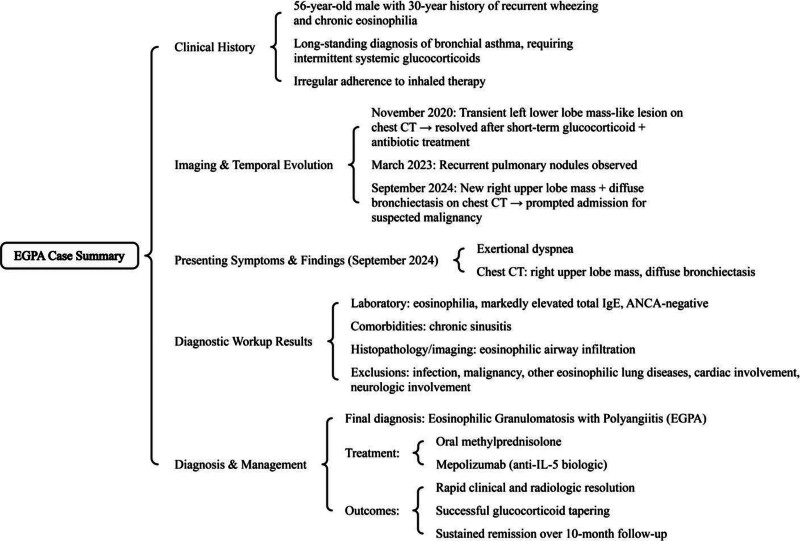
Diagnostic and therapeutic mind map of the case.

The patient was diagnosed with EGPA and initiated on combination therapy consisting of oral methylprednisolone (12 mg 3 times daily) and mepolizumab (300 mg). Significant clinical improvement was observed after 5 days, and the patient was subsequently discharged. Three weeks postdischarge, follow-up laboratory tests revealed a white blood cell count of 10.4 × 10^9^/L, absence of eosinophils (0%), total IgE level of 2680.89 kU/L, and IL-5 level of 0.42 pg/mL. One month after discharge, a chest CT scan demonstrated complete resolution of the right upper lobe lesion (Fig. 4B). The patient’s dyspnea had markedly improved, and the methylprednisolone dose was gradually tapered to 16 mg once daily. Mepolizumab was continued at standard intervals of every 4 weeks for a total of 6 doses. During the 10-month follow-up period, the patient remained free of dyspnea episodes, with no disease relapse and stable clinical condition. The diagnostic and therapeutic mind map of the case is shown in Figure 5.

## 3. Discussion

The patient had previously been misdiagnosed with asthma and failed to achieve adequate control of asthmatic symptoms. The development of pulmonary masses raised clinical suspicion of lung cancer. Following a comprehensive evaluation of the medical history and laboratory findings, EGPA was suspected and subsequently confirmed by the favorable response to targeted therapy. The diagnosis EGPA was supported by the following clinical and laboratory findings: migratory pulmonary masses; refractory asthma; persistent peripheral blood eosinophilia (≥1 × 10^9^/L), confirmed on multiple occasions; obstructive ventilatory dysfunction; chronic inflammatory involvement of multiple paranasal sinuses; and histopathological confirmation of eosinophilic infiltration in the interstitial tissue of the bronchial mucosa.

EGPA is a subtype of antineutrophil cytoplasmic antibody-associated vasculitis, distinct from granulomatosis with polyangiitis (GAP) and microscopic polyangiitis (MAP) in both clinical features and pathophysiology.^[1,2]^ First described by Churg and Strauss in 1951, EGPA typically progresses through overlapping phases, beginning with an asthma- and allergy-dominant prodromal phase, followed by eosinophilic tissue infiltration and, in some cases, systemic vasculitis.^[3–5]^ Based on ANCA status, EGPA is broadly classified into ANCA-positive, vasculitis-driven and ANCA-negative, eosinophil-driven phenotypes.^[6,7]^ Eosinophilic infiltration, granulomatous inflammation, and vasculitis may lead to multi-organ involvement, commonly affecting the lungs, sinuses, peripheral nerves, kidneys, heart, and skin.

The differential diagnosis included consideration of allergic bronchopulmonary aspergillosis (ABPA), lung cancer, GPA, and MPA. The clinical manifestations of ABPA are diverse and lack specificity. Common symptoms include cough, expectoration, wheezing, low-grade fever, emaciation, fatigue, and chest pain. Patients with ABPA and concomitant bronchiectasis may exhibit varying degrees of hemoptysis. In this case, the patient exhibited an absence of typical sputum plugs, hemoptysis, and detectable Aspergillus fumigatus in bronchoalveolar lavage fluid, normal levels of Aspergillus-specific IgE, and a negative galactomannan test result. Moreover, the presence of sinusitis was inconsistent with the clinical features of ABPA. Lung cancer was excluded based on the patient’s contrast-enhanced chest CT scan, which demonstrated no characteristic malignant imaging features, including lobulation, spiculation, or vascular clustering. Tumor marker analysis yielded normal results, and the resolution of pulmonary lesions following corticosteroid therapy further supported the absence of a malignant process. GPA, formerly referred to as Wegener granulomatosis, predominantly affects small arteries, veins, and capillaries, with the respiratory tract and kidneys being the most frequently involved organ systems. However, this condition is not typically associated with wheezing or marked peripheral blood eosinophilia. Moreover, GPA is serologically defined by the presence of cytoplasmic antineutrophil cytoplasmic antibodies and anti-proteinase 3 antibodies, which were not detected in the present case. MPA is a systemic necrotizing vasculitis that predominantly affects small blood vessels, particularly microarterioles, capillaries, and venules within organs such as the kidneys, skin, and lungs. It typically presents with necrotizing glomerulonephritis and pulmonary capillaritis. In contrast to EGPA, MPA is not associated with wheezing or significant peripheral blood eosinophilia. Furthermore, MPA is characterized by frequent positivity for perinuclear antineutrophil cytoplasmic antibodies and anti-MPO antibodies, while histopathological findings generally show no eosinophilic infiltration or granulomatous lesions.

In addition, this case requires careful differentiation from other eosinophilic disorders, particularly hypereosinophilic syndrome (HES) and eosinophilic pneumonia. Although the patient had long-standing eosinophilia and markedly elevated IgE levels, HES is unlikely. The clinical course was dominated by a 30-year history of asthma with recurrent wheezing, which is atypical for HES. In addition, there was no evidence of eosinophil-mediated multi-organ damage, and eosinophil counts were variable and responsive to glucocorticoids and anti-IL-5 therapy. The presence of asthma, chronic sinusitis, bronchiectasis, and steroid-responsive migratory pulmonary lesions further supports an EGPA-spectrum disorder. Eosinophilic pneumonia was also considered but is unlikely, as bronchoalveolar lavage fluid showed only 5% eosinophils, and imaging revealed recurrent, migratory, mass-like lesions rather than diffuse or peripheral consolidations. The association with long-standing asthma, sinonasal disease, and the excellent response to mepolizumab further argue against eosinophilic pneumonia.

EGPA is a rare disease with an annual incidence of approximately 0.5 to 4.2 cases per million and a prevalence of 10 to 14 per million, with no clear sex predilection.^[8,9]^ It comprises 2 main immunophenotypes: an ANCA-positive, vasculitis-driven phenotype and an ANCA-negative, eosinophil-driven phenotype. ANCA is detected in about 30% to 40% of patients with EGPA, with most cases directed against MPO rather than PR3. The presence of ANCA contributes to neutrophil activation, resulting in the release of reactive oxygen species, cytotoxic enzymes, and the formation of neutrophil extracellular traps, all of which are implicated in the pathogenesis of vascular injury.^[10]^ In ANCA-negative EGPA, T-helper type 2 (Th2) cells and group 2 innate lymphoid cells (ILC2s) play a central role in disease pathogenesis. Interleukin-5 (IL-5), which is secreted by Th2 cells and ILC2s in response to IL-33 during activation of the innate immune system, promotes eosinophil maturation, proliferation, and extravasation. Following extravasation, eosinophils are recruited to sites of inflammation through the synergistic effects of IL-5 and eosinophil-specific chemokines. Upon reaching the target tissue, eosinophils undergo degranulation, thereby contributing to eosinophilic inflammation.^[11–13]^ Clinically, ANCA-positive disease more often presents with renal involvement and peripheral neuropathy, whereas ANCA-negative disease is associated with eosinophil-related organ injury, particularly affecting the lungs and heart.^[14,15]^ Cardiac involvement is linked to a worse clinical prognosis.^[16]^ Furthermore, certain forms of organ damage, including cutaneous lesions, gastrointestinal involvement, and central nervous system abnormalities, may develop independently of ANCA status.^[17–19]^ In this patient, long-standing, relatively stable asthma with intermittent eosinophilia and repeated short-term responses to glucocorticoids likely masked disease evolution and delayed recognition of EGPA. This course is consistent with an asthma-dominant, ANCA-negative phenotype, in which extrapulmonary vasculitic manifestations are often mild, late-onset, or absent. Recurrent steroid-responsive migratory pulmonary lesions and chronic sinonasal disease emerged only later, prompting diagnostic reconsideration, while repeated glucocorticoid exposure may have further suppressed overt vasculitic features.

Currently, no universally accepted diagnostic criteria for EGPA exist; however, 2 widely used classification systems are available: the 1990 ACR criteria and the updated 2022 ACR/EULAR criteria, both with high sensitivity and specificity. The 1990 ACR classification criteria include 6 clinical and histopathological parameters, at least 4 of which must be met for a patient to be classified as having EGPA: peripheral blood eosinophilia (>10%); asthma; pulmonary infiltrates; sinusitis; mononeuropathy or polyneuropathy; and extravascular eosinophilic infiltration on biopsy.^[1]^ In contrast, the 2022 ACR/EULAR criteria (Table 1) apply a weighted scoring system incorporating clinical, laboratory, and histopathological features, with a total score ≥ 6 supporting EGPA classification after exclusion of mimicking conditions.^[20]^ The diagnostic criteria from the 2017 MIRRA trial may inform EGPA definition and include asthma, eosinophilia, and at least 2 of the following: eosinophilic vasculitis, perivascular eosinophilic infiltration, or eosinophil-rich granulomatous inflammation on histology; neuropathy; pulmonary infiltrates; sinus abnormalities; cardiomyopathy; glomerulonephritis; alveolar hemorrhage; palpable purpura; or positive ANCA. However, these criteria are based on expert consensus and require prospective validation.^[21]^ Despite ongoing research efforts, no novel biomarkers – such as periostin or galectin-10 – have been validated for application in EGPA diagnosis or assessment of disease activity. Given the lack of universally accepted diagnostic criteria, the diagnosis of EGPA primarily derived from histopathological findings. Although tissue biopsy remains an essential diagnostic method, its sensitivity for detecting vasculitis and granulomatous lesions is relatively low. Consequently, clinical judgment plays an increasingly important role in preventing missed or incorrect diagnoses. Unfortunately, no pathological evidence of vasculitis was obtained in this case. One possible explanation is that the patient may have been in the transitional phase from the initial prodromal stage to the subsequent eosinophilic infiltration stage, during which overt vasculitis may not yet be apparent. The diagnosis could have been further confirmed had pathological evidence of vasculitis become available at a later stage. The clinical manifestations of this case were highly indicative of EGPA, and the marked improvement in pulmonary lesions following treatment provides strong support for the clinical diagnosis.

**Table 1:** EGPA diagnostic evidence and 2022 ACR/EULAR scores.

Diagnostic feature	Evidence	ACR/EULAR criteria score
A maximum eosinophil count ≥ 1 × 109/L (+5)	Present	Yes
Obstructive airway disease (+3)	Present	Yes
Nasal polyps (+3)	Present	Yes
Extravascular eosinophilic-predominant inflammation (+2)	Absent	No
Mononeuritis multiplex and/or motor neuropathy not due to radiculopathy (+1)	Absent	No
C-ANCA or anti PR3-ANCA positivity (−3)	Absent	No
Haematuria (−1)	Absent	No

ACR/EULAR = American College of Rheumatology/European Alliance of Associations for Rheumatology, C-ANCA = cytoplasmic antineutrophil cytoplasmic antibodies, EGPA = eosinophilic granulomatosis with polyangiitis, PR3 = proteinase 3.

The treatment of EGPA involves glucocorticoids, immunosuppressive agents, and biologics, tailored to disease severity, activity, and organ involvement. Disease severity is assessed using the Five-Factor Score (FFS), a validated tool from the French Vasculitis Study Group that includes 5 factors: age over 65, heart failure, severe gastrointestinal involvement, renal failure, and absence of ear, nose, and throat manifestations.^[22]^ Patients with an FFS of 0 usually receive glucocorticoid monotherapy, while those with an FFS ≥ 1 – indicating higher risk and worse outcomes – require combination therapy with glucocorticoids and immunosuppressants. Treatment is divided into 2 phases: remission induction and remission maintenance.^[14]^ Induction therapy for EGPA primarily consists of glucocorticoids and/or immunosuppressive agents, with cyclophosphamide being a commonly used option. For maintenance therapy, azathioprine or methotrexate is generally recommended following successful induction of remission. Beyond conventional therapies, biologics have expanded treatment options and improved outcomes. Key biologics include anti-IL-5/IL-5 receptor antibodies (e.g., mepolizumab, benralizumab) and anti-CD20 therapy, primarily rituximab.

In this case, the patient had no evidence of peripheral neuropathy, alveolar hemorrhage, or other life-threatening manifestations and was therefore classified as having active, non-severe EGPA according to the 2021 American College of Rheumatology/Vasculitis Foundation guidelines.^[23]^ In addition, the patient’s FFScore was 0, indicating a low-risk disease profile. Together, these assessments supported remission induction with glucocorticoids in combination with biologic therapy (mepolizumab) rather than cytotoxic immunosuppressants. Following 1 month of treatment, the corticosteroid dose was gradually reduced, which led to marked improvement in asthma symptoms and significant radiological resolution of pulmonary lesions, all within a favorable safety profile. Mepolizumab is a humanized monoclonal IgG1 antibody that selectively targets free IL-5, thereby blocking its binding to IL-5 receptors expressed on the surface of eosinophils. Clinical trials have demonstrated that mepolizumab significantly decreases eosinophil counts, increases the likelihood of achieving remission, prolongs remission duration, and is associated with a lower risk of relapse compared to placebo.^[21]^ Genetic variation in IL-5 is predominantly associated with ANCA-negative EGPA, indicating a potential for enhanced therapeutic response to mepolizumab within this patient subgroup. Nevertheless, further clinical evidence is required to confirm this hypothesis.^[7]^

Despite significant advances in the understanding of EGPA pathogenesis and improvements in its clinical management, the diagnosis and therapy in EGPA remain challenging for healthcare providers. It remains uncertain whether treatment should be stratified based on ANCA status. The optimal initial corticoster dose and the most appropriate tapering regimen have not yet been clearly established. Furthermore, whether mepolizumab can prevent long-term tissue damage and enhance the overall prognosis of EGPA requires further clinical investigation.

## Author contributions

**Data curation:** Wenhua Gu, Wei Yang.

**Investigation:** Wenhua Gu, Wei Yang, Chaomin Wu.

**Project administration:** Wenhua Gu, XiongBiao Wang.

**Resources:** XiongBiao Wang, Chaomin Wu.

**Writing** – **original draft:** Wenhua Gu.

**Writing** – **review & editing:** XiongBiao Wang.
